# Recent advances in exploring the composition and evolution of the prokaryotic selenoproteome

**DOI:** 10.1128/aem.00556-26

**Published:** 2026-05-04

**Authors:** Yan Zhang, Shuting Wang, Hengtao Li, Xuan Chen

**Affiliations:** 1 Shenzhen Key Laboratory of Marine Bioresources and Ecology, Brain Disease and Big Data Research Institute, College of Life Sciences and Oceanography, Shenzhen University 616964 , Shenzhen, Guangdong Province, People's Republic of China; 2 Shenzhen-Hong Kong Institute of Brain Science-Shenzhen Fundamental Research Institutions 644977 , Shenzhen, Guangdong Province, People's Republic of China; The Pennsylvania State University, University Park, Pennsylvania, USA

**Keywords:** evolution, bioinformatics, prokaryotes, selenoprotein, selenocysteine, selenium

## Abstract

Selenoproteins, a unique class of proteins critical for cellular antioxidant defense, are characterized by the incorporation of selenocysteine (Sec) in their active sites. Sec is co-translationally inserted into proteins via a specialized mechanism that reprograms the UGA codon to encode Sec, involving a specific RNA structure designated the Sec insertion sequence (SECIS) element and several essential enzymes. Although numerous selenoproteins have been identified in prokaryotes (primarily bacteria), the detection of selenoprotein genes in these organisms remains challenging, largely due to difficulties in distinguishing the Sec-encoding UGA codon from standard termination signals. In recent years, computational approaches for predicting selenoprotein genes, along with comparative genomic analyses of Sec-encoding machinery and selenoproteomes, have emerged as a promising and rapidly evolving field, offering new insights into Sec utilization in bacteria and archaea. This review provides a comprehensive overview of the latest advancements in the study of selenoproteins in prokaryotes. We summarize the molecular mechanisms underlying Sec biosynthesis and incorporation, and the structural diversity of SECIS elements in bacteria and archaea. We then describe current computational strategies for the identification of prokaryotic selenoprotein genes and present an updated, extensive catalog of prokaryotic selenoproteins documented to date, emphasizing those with well-established functions. Finally, we discuss recent progress in understanding the evolutionary dynamics of the Sec-encoding system and selenoproteins across prokaryotes, with a focus on the archaea-to-eukaryote transition of Sec machinery and selenoproteins. Overall, this review offers a unified perspective on the identification, functions, and evolution of selenoproteins in prokaryotes.

## INTRODUCTION

Selenium (Se) is an essential trace element that supports vital growth and developmental processes across all domains of life, from bacteria to humans (1, 2). Despite being present in trace amounts, this micronutrient is involved in a variety of biological functions. It primarily exists as selenocysteine (Sec), the 21st genetically encoded amino acid, which is co-translationally inserted into the active site of selenoproteins by recoding the UGA codon (3, 4). These specialized proteins participate in several critical cellular processes. In eukaryotes, selenoproteins play important roles in redox homeostasis, anti-inflammatory/antiviral defense, immune regulation, hormone metabolism, and reproductive functions, while in prokaryotes, they are mainly involved in anaerobic respiration, amino acid and coenzyme metabolism, as well as the maintenance of redox balance (4–7).

The biosynthesis of Sec and its incorporation into selenoproteins rely on a complex molecular machinery that comprises both common and unique components among the three domains of life (8–10). To date, a substantial number of selenoproteins have been identified in diverse prokaryotic and eukaryotic organisms, many of which were characterized using robust bioinformatics approaches (11–16). Notably, bacterial selenoprotein families are nearly three times as numerous as those in eukaryotes, highlighting their remarkable diversity in this domain. The selenoproteome (the complete set of selenoproteins in an organism) varies significantly across species. While the precise functions of most selenoproteins (particularly in bacteria) remain poorly understood, many are implicated in antioxidant defense mechanisms (17).

In certain prokaryotic organisms, Se is incorporated into 5-methylaminomethyl-2-selenouridine (mnm⁵Se²U, or SeU), a selenonucleoside located at the wobble position of anticodons in several tRNAs that regulates translational processes, and into a Se-containing cofactor used by specific molybdoenzymes (18, 19). Recently, two novel selenometabolites, selenoneine and ovoselenol, have also been characterized in diverse bacterial species (20, 21). In addition, due to the close chemical similarity between Se and sulfur (S), Se can be metabolized via S assimilation pathways, leading to the nonspecific incorporation of Sec in place of Cys in proteins. However, this process often requires higher Se concentrations and may result in proteins where Sec is misplaced or non-functional. Such misincorporation generally provides no biological advantage and can even be harmful or neutral in effect. Consequently, these proteins are excluded from the selenoproteome, which is defined by the presence of Sec in functionally critical sites mediated by dedicated biosynthetic machinery.

Previous studies have thoroughly explored and characterized the types, functions, and evolutionary trajectories of selenoproteins in different eukaryotic species, with comprehensive summaries provided in a series of reviews (6, 10, 14, 22–25). In contrast, research on selenoproteins in bacteria and archaea has lagged far behind that in eukaryotes. Over the past several years, breakthroughs in high-throughput sequencing technologies have enabled the generation of genomic sequences for diverse prokaryotic species. Moreover, the rapid development and widespread application of advanced bioinformatics strategies and methods have facilitated genome-scale computational and comparative analyses of selenoproteins and selenoproteomes across a wide range of prokaryotes. These achievements have significantly improved our understanding of Se utilization, biological roles, and evolutionary dynamics in bacteria and archaea.

In this review, we mainly focus on recent advances in the identification of selenoproteins in bacteria and archaea, along with their evolutionary trends in prokaryotes, to offer a comprehensive and integrated view of Se utilization and evolution in these domains.

## Se UPTAKE, Sec BIOSYNTHESIS, AND INCORPORATION INTO PROTEINS IN PROKARYOTES

Se exists in nature in both inorganic and organic forms. The inorganic forms include elemental Se, selenide, selenite, and selenate, whereas the primary organic forms are Sec and selenomethionine (SeMet) (26). Although the exact mechanisms of microbial Se uptake are still not fully understood, emerging evidence indicates that Se may utilize the S metabolic pathways, which can be taken up, in the form of selenite/selenate, by the sulfate transport system and then reduced to selenide via the assimilatory sulfate reduction system (27, 28). Additionally, phosphate transporters have been implicated in selenite uptake and biotransformation in plants, yeasts, and bacteria (29, 30). However, a specific high-affinity Se transport system has not been identified in prokaryotes.

**Fig 1:**
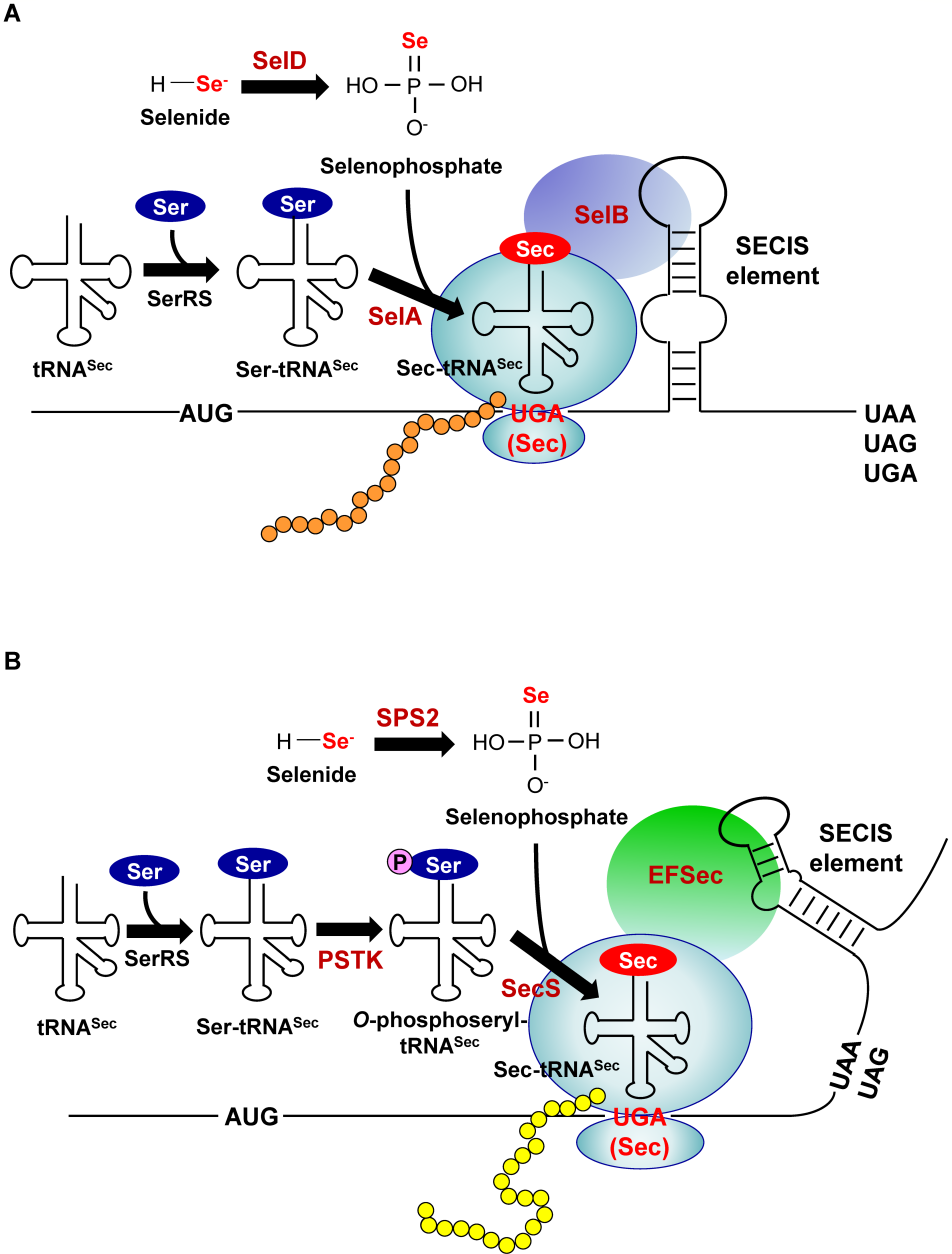
A general scheme of Sec biosynthesis and insertion into proteins in prokaryotes. (**A**) Bacteria: SelA, bacterial Sec synthase; SelB, bacterial Sec-specific elongation factor; SelD, selenophosphate synthetase; SerRS, seryl-tRNA synthetase. (**B**) Archaea: SPS2, selenophosphate synthetase 2; SecS, archaeal/eukaryotic Sec synthase; EFSec, archaeal/eukaryotic Sec-specific elongation factor; PSTK, *O*-phosphoseryl-tRNA^Sec^ kinase. Specific proteins involved in Sec synthesis and incorporation into selenoproteins are highlighted in brown.

The molecular mechanisms underlying Sec biosynthesis and incorporation into selenoproteins in prokaryotes have been thoroughly summarized in previous reviews (8, 31–33). In bacteria, this process requires a UGA codon encoding Sec (Sec-UGA), a Sec insertion sequence (SECIS) element (a *cis*-acting stem-loop structure immediately downstream of the Sec-UGA codon), tRNA^Sec^ (a specific tRNA with an anticodon complementary to UGA, encoded by the *selC* gene), and several *trans*-acting factors dedicated to Sec incorporation (Fig. 1A). The tRNA^Sec^ is initially aminoacylated with serine to form seryl-tRNA^Sec^ by canonical seryl-tRNA synthetase (SerRS) and then converted to selenocysteyl-tRNA^Sec^ (Sec-tRNA^Sec^) by Sec synthase (SelA). SelA uses selenophosphate as the Se donor, which is synthesized from selenide and ATP by selenophosphate synthetase (SelD, or named SEPHS2 in eukaryotes). During translation, the Sec-specific elongation factor SelB binds the SECIS element, forming a quaternary complex with Sec-tRNA^Sec^ and GTP. This complex translocates to the ribosome, where the lower helical region of the SECIS element undergoes unwinding. Upon arrival of the UGA codon at the ribosomal A site, SelB interacts with the ribosome to position charged Sec-tRNA^Sec^ into the A site. Following peptide bond formation and ribosomal translocation, the SECIS element refolds, enabling its reuse in assembling new quaternary complexes for subsequent rounds of UGA decoding by other ribosomes. In certain bacterial lineages, such as *Alphaproteobacteria*, *Gammaproteobacteria*, and *Nitrospirae*, the *selC* gene was found to be either entirely embedded within or partially overlaps with the *selB* gene, implying a novel mechanism for maintaining homeostasis between SelB and tRNA^Sec^, as well as for controlling the expression level of *selB* in these bacteria (34).

While archaea and eukaryotes share a conserved core mechanism with bacteria for the biosynthesis of Sec and its incorporation into selenoproteins, they require additional enzymatic steps and components, including archaeal/eukaryotic Sec synthase (SecS), Sec-specific elongation factor EFSec (a SelB homolog with a conserved N-terminal region but different C-terminal domains among bacteria, archaea, and eukaryotes), and *O*-phosphoseryl-tRNA^Sec^ kinase (PSTK) (32, 35). Unlike bacteria, where SECIS elements reside within coding regions, SECIS elements in archaea and eukaryotes are both located in the 3′-untranslated regions (3′-UTRs) of selenoprotein genes. Although the eukaryotic Sec-encoding system is generally thought to have evolved from archaeal ancestors, there are clear distinctions between them. Archaea lack several eukaryotic-specific proteins essential for Sec incorporation, such as SECIS-binding protein 2 and tRNA selenocysteine 1-associated protein 1, suggesting somewhat divergent mechanisms between the two domains of life (36). Furthermore, archaeal SECIS elements exhibit distinct sequence and structural features compared to their bacterial and eukaryotic counterparts (37–39). A general scheme of Sec biosynthesis in archaea is illustrated in Fig. 1B.

Certain eukaryotic microorganisms, such as yeasts, exhibit an inherent ability to accumulate and biotransform Se into various chemical forms, including Sec (28, 40). In these organisms, Sec can be nonspecifically synthesized through the metabolic conversion of SeMet via the S assimilation pathway enzymes, such as cystathionine β-synthase and cystathionine γ-lyase, which normally process S-containing amino acids but can also accommodate Se analogs due to chemical similarities between Se and S (28). However, it is uncertain whether prokaryotes employ a SeMet-to-Sec conversion pathway similar to that observed in yeasts. Further comparative studies on Se metabolism across different domains of life are needed to address this knowledge gap.

It has been reported that Se recycling from Sec is primarily mediated by Sec lyase, which catalyzes the reduction of Sec to selenide in the presence of reducing agents (41). The produced selenide is subsequently converted to selenophosphate by SelD for selenoprotein biosynthesis. However, these Sec lyases belong to the cysteine (Cys) desulfurase superfamily and demonstrate restricted substrate specificity for Se metabolism, as they predominantly participate in S metabolic pathways by mobilizing S from Cys for various processes (42). On the other hand, a distinct Sec lyase that specifically decomposes Sec into alanine and selenide has been identified in animals but remains ambiguous in bacteria and archaea (43). A recent study has identified a candidate Sec lyase (named SclA) in *Enterococcus faecalis*, which is encoded by a gene in a *selD*-containing operon potentially dedicated to Se metabolism (44). While SclA shows low catalytic activity with Cys as a substrate, its enzymatic preference is significantly biased toward Sec over Cys, supporting its primary role in Sec catabolism.

## PROKARYOTIC SECIS ELEMENTS

As previously discussed, all selenoprotein genes are characterized by the presence of both a Sec-UGA codon and a downstream SECIS element. The SECIS element is an essential and highly specific RNA structure that directs the incorporation of Sec into the nascent polypeptide chain during translation. Its conserved sequence and structural features can be utilized for reliable computational prediction (45).

**Fig 2:**
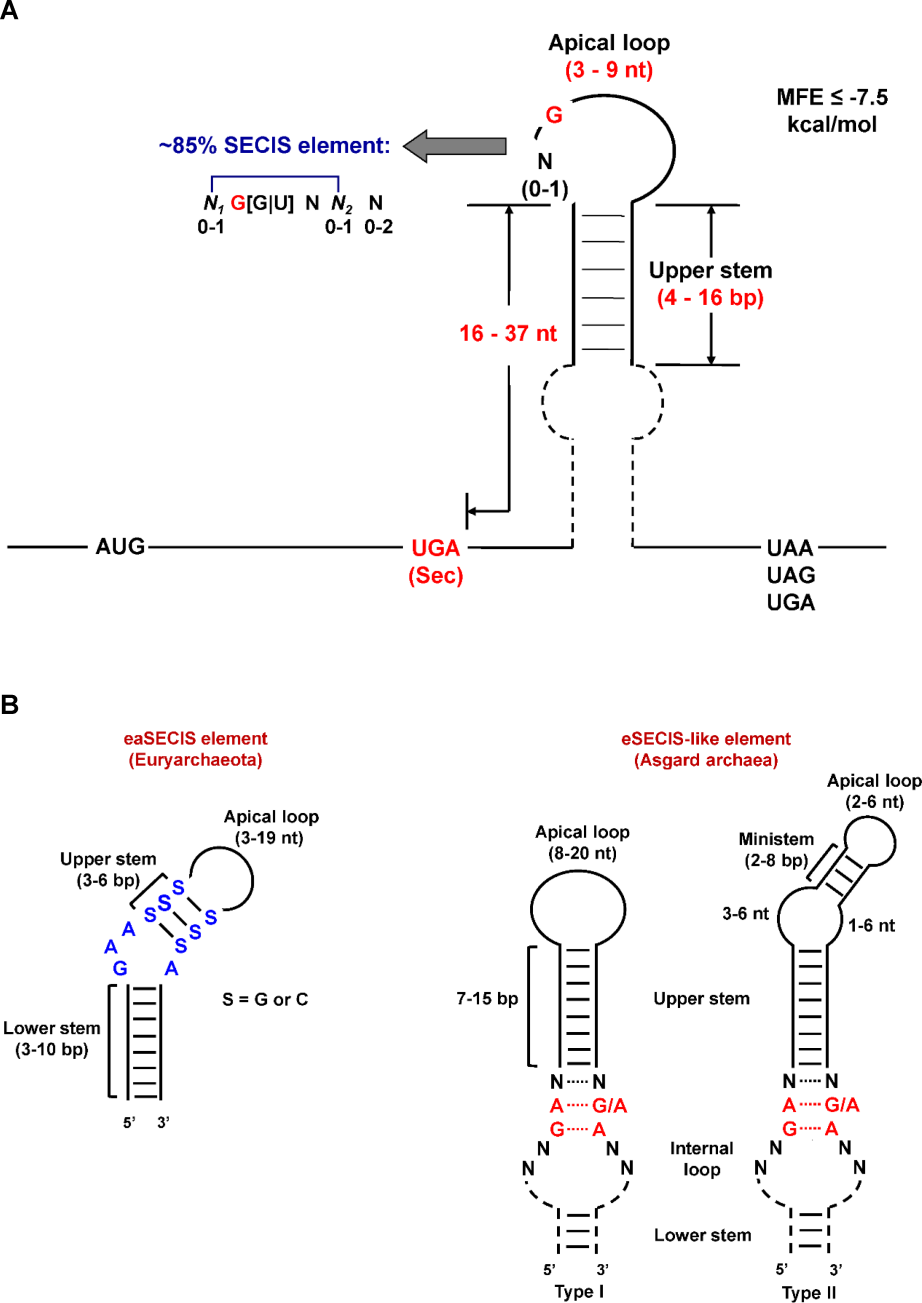
Consensus structural models of prokaryotic SECIS elements. (**A**) Bacteria. The Sec-UGA codon is highlighted in red. The bacterial SECIS model includes the following constraints: (i) a 3–9 nt apical loop and a 4–16 bp upper stem; (ii) at least one guanosine (G) in the first two positions in the apical loop (mostly contain the N_1_(0-1)G[G|U]NN_2_(0-1)N(0-2) pattern in which N_1_ and N_2_ form a base pair); (iii) a spacing of 16–37 nt between the Sec-UGA codon and the apical loop; and (iv) minimum free energy (MFE) ≤ −7.5 kcal/mol. (**B**) Archaea. Both the eaSECIS and eSECIS-like (type I/II) models are depicted, with the allowed length ranges for their respective stems and loops clearly indicated. Conserved nucleotides are highlighted in different colors, including the non-Watson-Crick base pair quartet in the eSECIS-like model, as well as the GAA_A bulge and S-S base pairs in the eaSECIS model. N represents any nucleotide.

The bacterial SECIS (bSECIS) element is typically located immediately downstream of the Sec-UGA codon in the coding regions of selenoprotein mRNAs. Although the best characterized bSECIS elements are found in genes encoding three formate dehydrogenases (FDHs) in *Escherichia coli* (46), many putative bSECIS elements identified in other bacterial selenoprotein mRNAs bear no resemblance to each other or to their *E. coli* counterparts. Based on computational analyses of predicted bSECIS elements in a variety of bacterial selenoprotein genes, a consensus structural model representing the common stem-loop core was proposed (13). This model reveals that a single guanine (G), often followed by another G or uracil (U), occupies one of the first two positions within a small apical loop of the bSECIS structure and that the distance between Sec-UGA and the apical loop ranges from 16 to 37 nucleotides for most bSECIS elements (Fig. 2A). These findings suggest that the majority of bSECIS elements can be described by a conserved structural framework. However, putative bSECIS elements identified in a subset of selenoprotein genes fail to satisfy the constraints for this model, indicating the presence of distinct classes of bSECIS elements in bacteria.

In archaea, SECIS elements are most often located in the 3′-UTR of selenoprotein genes. Early studies revealed that archaeal SECIS elements diverge significantly from their eukaryotic counterparts (12). Eukaryotic SECIS (eSECIS) elements typically consist of two helices separated by an internal loop, a SECIS core structure with an unusual 5′-GA_GA-3′ non-Watson-Crick base pair quartet at the upper stem base (preceding the first GA is always AU (rarely GU) to form the AUGA tetranucleotide motif), and an apical loop mostly containing an unpaired AAR motif (where R = purine) (47, 48). The eSECIS elements can be classified as type I or type II based on the presence of an additional apical ministem following the AAR motif, found in type II only. In contrast, archaeal SECIS elements identified in *Euryarchaeota* (designated eaSECIS hereafter) possess two stems separated by an internal bulge with a conserved purine-only 5′-GAA_A-3′ motif, three consecutive C-G or G-C (or S-S, where S = C or G) base pairs in the upper stem, and an apical loop of variable length, all of which differ markedly from eSECIS elements (12, 37, 38). By employing a newly developed reporter system designed to monitor Sec insertion in the archaeon *Methanococcus maripaludis*, Peiter et al. identified a minimal eaSECIS element required for Sec incorporation and demonstrated that a conserved structural motif, especially two invariant adenines (AA) within the GAA_A motif, is critical for its function (49, 50). In recent years, a novel archaeal superphylum, the Asgard archaea, has been discovered, which harbors numerous eukaryotic-like features and is considered the closest archaeal relatives of eukaryotes (51, 52). Previous studies have reported that several Asgard archaea (such as *Lokiarchaeota* and *Thorarchaeota*) possess both Sec-encoding machinery and conserved RNA structures similar to eSECIS elements, suggesting the existence of intermediate forms between archaeal and eukaryotic Sec insertion systems (53–55). A general model for the key regions of eSECIS-like structures identified in Asgard archaea was recently proposed, including two types: type I (without an apical ministem) and type II (with an apical ministem) (56). Consensus models for eaSECIS and eSECIS-like structures are shown in Fig. 2B.

Although the SECIS element plays a crucial role in facilitating the insertion of Sec into selenoproteins in prokaryotes, alternative SECIS-independent strategies for Sec incorporation, particularly through protein engineering, have been actively explored. Arnér et al. demonstrated that, via a recombinant selenoprotein production system, Sec can be efficiently incorporated at a predefined UAG codon, with the SECIS element no longer being an absolute necessity (57). Additionally, the redesign of SECIS-independent variants of the *selB* gene represents another promising approach to maintaining the natural efficiency and specificity of Sec incorporation in bacteria (33, 58).

## *IN SILICO* IDENTIFICATION OF SELENOPROTEINS IN PROKARYOTES

The UGA codon mainly functions as a canonical translational termination signal, but its dual role as a Sec-encoding codon in selenoprotein genes poses a major challenge for genomic annotation. This ambiguity frequently causes automated annotation tools to misidentify selenoprotein genes as truncated proteins (via premature termination) or to entirely overlook them (false negatives). Current genome annotation pipelines, which rely solely on standard stop codons (UAA, UAG, and UGA), are particularly error-prone in Sec-utilizing organisms. Thus, despite the exponential growth of genomic data, accurately annotated selenoprotein genes remain rare, especially in prokaryotes.

Over the past two decades, a significant number of selenoprotein genes have been identified in diverse bacterial organisms, primarily through bioinformatics-driven approaches. These computational algorithms have proven highly effective in uncovering novel selenoprotein families, enabling large-scale exploration of both genomic and metagenomic data sets. Two primary strategies, SECIS-dependent and SECIS-independent methods, have been developed for selenoprotein gene prediction (15, 16).

The SECIS-based approach for selenoprotein gene prediction involves a three-step strategy: (i) identifying potential SECIS elements by screening for conserved primary and secondary structural features that match the bSECIS model; (ii) analyzing the genomic context to identify suitable protein-coding regions; and (iii) selecting high-confidence candidates through further analysis. A program named bSECISearch was developed to predict bSECIS elements and selenoprotein genes in bacterial genomes (13).

Considering that nearly all selenoproteins have Cys-containing homologs (where Sec is replaced by Cys), SECIS-independent approaches have been developed to predict selenoprotein genes using a Cys/Sec pair-based strategy (12, 59–62). With a predefined set of Cys-containing proteins as query sequences, a tblastn-based search is performed to identify TGA-containing nucleotide sequences whose translated products exhibit homology to the queries, with conserved Cys residues in the query proteins aligning to translated TGA codons (forming Cys/Sec pairs). To minimize false positives, additional filters are applied, such as the presence of potential bSECIS elements and the detection of TGA-containing homologs in other organisms. These criteria enhance the accuracy of novel selenoprotein gene discovery. Haft et al. employed a related strategy to identify new selenoprotein candidates (63, 64). Their approach utilized multiple sequence alignments of groups of proteins, focusing on cases where unexpected N- or C-terminal truncations resulted in extended translational regions containing UGA codons. When these UGA codons aligned with Cys residues in nonselenoprotein homologs, they were considered as potential Sec recoding sites.

By using these established methods, a large number of novel selenoproteins have been successfully identified in both fully sequenced bacterial genomes and environmental metagenomes (12, 13, 59–64). However, despite these achievements, both SECIS-dependent and SECIS-independent strategies have significant limitations. For example, they are highly time- and resource-intensive, prone to a relatively high rate of false positives during the selection of new selenoprotein candidates, and may overlook a certain subset of known selenoproteins. Given the exponential growth of bacterial genomic data, there is an urgent need for rapid and efficient algorithms capable of accurately annotating selenoproteomes in sequenced bacteria.

In recent years, deep learning techniques have achieved remarkable advancements across diverse biological research fields, including the study of trace elements. A deep learning-based algorithm, deep-Sep (available at http://deepsep.metalbioinfolab.net:7001/), was developed for fast and accurate prediction of selenoprotein genes in bacteria, demonstrating superior performance in comparison to existing methods (65). This algorithm is designed for routine investigations of bacterial genomes for the identification of selenoprotein genes. It employs a Bidirectional Encoder Representations from Transformers (BERT)-based deep neural network to generate an optimal model for Sec-UGA codon, and a homology search-based filtering step to remove false positives. When tested on independent bacterial genomes, deep-Sep not only identified all previously known selenoprotein genes but also discovered several new selenoprotein families. Very recently, using this method, Deng et al. conducted an extensive examination of over 44,000 bacterial and archaeal metagenome-assembled genomes (MAGs) from groundwater ecosystems and identified numerous selenoprotein sequences in diverse prokaryotic lineages, including potentially novel candidate selenoproteins that require further validation (66). These preliminary findings suggest that deep learning-based methodologies may offer a powerful and efficient approach for characterizing selenoproteomes in the rapidly expanding microbial genomic and metagenomic data sets.

Both SECIS-dependent and SECIS-independent approaches were previously used to predict archaeal selenoprotein genes in genomic databases (12). However, due to the restricted utilization of Sec in archaea, only a small number of selenoproteins could be identified in this domain.

Over the past few years, AlphaFold and its successive iterations, AlphaFold 2 and AlphaFold 3, have been widely adopted for predicting protein structures with unprecedented accuracy (67, 68). These AI-driven tools have revolutionized structural biology by enabling high-resolution modeling of protein structures, accurate prediction of protein-ligand interactions, and dynamic characterization of conformational transitions. To date, the applications of these tools have primarily focused on predicting the 3D structures of human selenoproteins and newly identified selenoproteins in specific bacteria such as *Clostridium difficile* (63, 69). In addition, they have also been utilized to elucidate the interactions between specific human selenoproteins (e.g*.*, selenoprotein M) and other biomolecules (70). However, the broader potential of these AI tools for prokaryotic selenoprotein structure prediction remains largely unexplored, especially when considering the diverse range of prokaryotic species and selenoprotein families. A systematic application of these approaches to predict selenoprotein structures across prokaryotic taxa might uncover conserved structural features, particularly in the regions surrounding the Sec residue. Such efforts would not only advance our understanding of selenoprotein structure-function relationships but also provide validation benchmarks to enhance confidence in selenoprotein predictions.

On the other hand, since tRNA^Sec^ is essential for selenoprotein biosynthesis, its efficient detection can facilitate the exploration of unknown selenoproteins in organisms lacking previously known selenoproteins but possessing tRNA^Sec^. The Secmarker tool was developed to accurately identify tRNA^Sec^ in genomic sequences, thereby advancing the discovery of novel prokaryotic selenoprotein families (71).

## PROKARYOTIC SELENOPROTEINS

To date, more than 110 selenoprotein families have been experimentally or computationally identified in prokaryotes (predominantly in bacteria). While several prokaryotic selenoprotein families, such as SelD/SEPHS2, glutathione peroxidase (GPX), deiodinase-like protein, selenoprotein W(SELENOW)-like protein, and methionine sulfoxide reductase A (MsrA), are also present in eukaryotes, the majority remain exclusive to bacteria (15, 16). Compared to bacteria, archaeal selenoproteins exhibit a highly restricted taxonomic distribution, with only a few selenoprotein families identified in specific methanogenic lineages (*Methanococcales* and *Methanopyrales*) (72). Notably, recent studies have revealed that certain Sec-utilizing Asgard archaea (e.g., *Lokiarchaeota* and *Borrarchaeota*) have uncovered homologs of bacterial selenoprotein families, suggesting a more complex evolutionary landscape of Sec utilization in archaea than previously recognized (56).

The majority of characterized selenoproteins contain a thioredoxin (Trx)-like fold with a redox-active motif. Despite the rapid expansion of genomic and metagenomic data sets in recent years, which has enabled the discovery of a variety of novel selenoprotein genes across diverse prokaryotic lineages, a systematic compilation of these proteins remains absent. Here, we present the most comprehensive inventory of prokaryotic selenoproteins to date by integrating experimentally validated and computationally predicted high-confidence candidates from the literature (Table 1) (12, 13, 59–66, 71–90). Selenoproteins sharing identical domain architectures but differing in Sec positions or Sec-related motifs are classified as distinct subfamilies. In this review, a total of 119 selenoprotein families/subfamilies are encompassed, with the vast majority featuring a single Sec residue, while a small subset contains 2–3 Sec residues. A substantial number of selenoproteins have been annotated in the NCBI protein databases, including RefSeq and GenBank. When researchers aim to retrieve non-redundant sequence data for specific selenoprotein families from RefSeq, for instance, the HesB-like protein family, they can employ targeted search queries in NCBI’s Protein portal with the syntax: selenocysteine [Properties] AND HesB-like AND WP_000000000:WP_999999999 [Accession]. Although the functions of most selenoproteins are not clear, many of them are either homologous to thiol-based oxidoreductases or contain redox-related motifs (e.g., UxxC and CxxU, where x denotes any amino acid). It is likely that the majority of these uncharacterized selenoproteins serve redox functions.

**TABLE 1:** Currently known and predicted selenoprotein families/subfamilies in prokaryotes

Selenoprotein family or subfamily	Domain ID (Interpro-based)	Sec-related motif (U: Sec)	No. of Sec	Representative sequence (accession number)	Ref.
Experimentally verified					
Formate dehydrogenase alpha subunit^*a*^	TIGR01553	UHxP	1	WP_010904702.1	(73)
Formylmethanofuran dehydrogenase subunit B^*a*^	TIGR03129	UHxP	1	CAA67419.1	(74)
Selenophosphate synthetase^*a*^	TIGR00476	UxxK	1	WP_083774555.1	(75)
Coenzyme F420-reducing hydrogenase alpha subunit^*a*^	TIGR03295	UxxC	1	WP_083774535.1	(76)
Methylviologen-reducing (or F420-non-reducing) hydrogenase alpha subunit (MvhA/VhuA/VhuU)^*a*^	NF041786	UxxC	1	P0C1V6.2	(77)
Coenzyme F420-reducing hydrogenase delta subunit (or F420- non-reducing hydrogenase delta subunit MvhD/VhuD)^*a*^	PF02662	CxxU	2	WP_012980414.1	(78)
Heterodisulfide reductase alpha subunit^*a*^	NF040770	CxxU	1	WP_162484757.1	(79)
HesB-like protein^*a*^	TIGR01911	UxGP	1	WP_083774540.1	(80)
Glycine reductase complex selenoprotein A	PF04723	CxxU	1	WP_079747582.1	(81)
Glycine reductase complex selenoprotein B	PF07355	UxxC	1	WP_246895825.1	(81)
D-proline reductase	TIGR04483	UxxC	1	WP_079281142.1	(82)
Peroxiredoxin (Prx)	PF00578	TxxU	1	WP_011365628.1	(83)
Thioredoxin (Trx)	PF00085	UxxC	1	WP_010956703.1	(84)
Glutaredoxin (Grx)	PF00462	UxxC	1	WP_010943784.1	(85)
Methionine sulfoxide reductase A	PF01625	–^*b*^	1	MBI4965933.1	(86)
Arsenite methyltransferase	NF008823	UxxG	1	WP_011987699.1	(87)
Predicted with high confidence					
Prx-like thiol:disulfide oxidoreductase^*a*^	PF00578	UxxC, UxxU	1~2	WP_010940744.1	(12)
Thiol:disulfide interchange protein	PF13098	UxxC	1	WP_011366075.1	(12)
Selenoprotein W (SELENOW)-like protein	PF10262	CxxU	1	AOH51717.1	(12)
Glutathione peroxidase (GPX)-like protein	PF00255	UxxT	1	WP_010957027.1	(12)
Homolog of AhpF N-terminal domain (Grx-like domain protein)	TIGR02187	UxxC	1	ABB15282.1	(12)
DsbG-like protein	NF041057	UxxC	1	WP_339958211.1	(12)
Fe-S oxidoreductase-like protein	PTHR43551	UGH	1	WP_174406253.1	(12)
DsrE-like protein	PF02635	UxxC	1	WP_014524487.1	(12)
FAD-dependent oxidoreductase (CoA-disulfide reductase)	TIGR03385	UGxP	1	WP_011365774.1	(12)
Distant alkylhydroperoxidase (AhpD) homolog	NF041047	CxxU	1	WP_255296099.1	(12)
Radical SAM domain protein	TIGR04167	GxxU	1	AAR34688.1	(13)
Rhodanese-like domain-containing protein form 1	PF00581	UxxS	1	WP_010941598.1	(13)
Rhodanese-like domain-containing protein form 2 (rhodanese-related sulfurtransferase)	PF00581	UxxG	1	MBM9537886.1	(13)
Rhodanese-like domain-containing protein form 3	NF041203	CxU	1	TKB26178.1	(13)
AhpD-like protein	TIGR01926	CxxU	1	MCB9421940.1	(59)
Arsenate reductase	TIGR00014	UxxS	1	MBT3519430.1	(59)
Molybdopterin-synthase adenylyltransferase MoeB	PF00899	–	1	MBT7809913.1	(59)
DsbA-like protein form 1^*a*^	PF01323	UxxC	1	NIP15863.1	(59)
Glutathione S-transferase (GST)-like protein	PF13409	–	1	MEK9676863.1	(59)
Deiodinase-like protein	PF00837	UxxC	1	MFL2763324.1	(59)
Thiol-disulfide isomerase-like protein	cd02966	UxxC	1	MGB1650493.1	(59)
Carboxymuconolactone decarboxylase (CMD)-like protein	PF02627	CxxU	1	MBW1767730.1	(59)
AhpD-related protein (hypothetical protein 1, Sargasso Sea metagenome)	NF041238	CxxU	1	MEE2999090.1	(59)
OsmC-like protein	PF02566	UxxT	1	MCS5647933.1	(59)
Rhodanese-like domain-containing protein form 4 (rhodanese-related sulfurtransferase)	PF00581	UxxG	1	MCY3690685.1	(59)
NADH:ubiquinone oxidoreductase subunit E	PF02508	–	1	–	(59)
Putative mercuric transport protein	PF02411	C/SxxU	1	ABB16073.1	(60)
Cation-transporting ATPase E1-E2 family	TIGR01525	UxxC	1	ABB15669.1	(60)
Methylated-DNA-protein-cysteine methyltransferase	PF01035	–	1	ABB14497.1	(60)
UGSC-containing protein	NF041046	UGSC	1	ABI76733.1	(60)
DUF3179 domain-containing protein	PF11376	UxxC/T	1	MDJ0986319.1	(60)
YHS domain-containing protein	NF041384	GxU	1	MDJ0831626.1	(60)
C-GCAxxG-C-C family protein form 1 (putative redox-active protein)	PF09719	–	1	MGD8741185.1	(60)
DUF166 domain-containing protein	NF041372	–	1	MDJ0831378.1	(60)
DUF1573 domain-containing protein	PF07610	UGC	1	MDJ0815992.1	(60)
Dual CXXC motif small protein (hypothetical protein OS_HP3)	NF041197	RxU	1	WP_425411677.1	(60)
Metal-binding protein (putative mercuric reductase)	NF041115	UxxU	2	MEE9531371.1	(60)
CYCXC family protein (hypothetical protein OS_HP4)	PF13798	UxxC	1	MFC1881021.1	(60)
Cobalamin synthesis CobW-related selenoprotein	NF041053	UxxC	1	MDH3886304.1	(60)
Peroxiredoxin-like family protein (AhpC/TSA family protein)	cd02970	UxxS	1	MDX2448127.1	(60)
VPGUxxT family thioredoxin-like protein (hypothetical protein OS_HP5)	NF041383	VPGU	1	WP_267462213.1	(60)
Distant Grx-like protein 1 (UXX-star selenoprotein family 1)	NF041114	UxxT	2	WP_258313609.1	(61)
Arsenate reductase-like protein	NF041106	UxxC	1	MDE3003982.1	(61)
Fe-S cluster domain-containing protein	NF005503	UxxC	1	WP_258165141.1	(61)
(2Fe-2S)-binding protein (copper chaperone Copz family) form 1	cd10141	GxUC	1	WP_245779778.1	(61)
(2Fe-2S)-binding protein (copper chaperone Copz family) form 2 (Csac_0668 family 2Fe-2S cluster-binding selenoprotein)	cd10141	CU	1	WP_346914553.1	(61)
CC/Se motif family selenoprotein (hypothetical protein predicted in Moorella thermoacetica)	NF041239	CxxU	1	WP_265736915.1	(61, 62)
MerB-like organometallic lyase SaoL (alkylmercury lyase MerB-like protein)	NF040728	CU	1	WP_238493467.1	(61, 63)
DUF1858 domain-containing protein	PF08984	CxxU	1	WP_012065717.1	(61, 62)
Proline reductase-associated electron transfer protein PrdC form 1	TIGR04481	CxxU	1	WP_243183503.1	(61, 88)
Proline reductase-associated electron transfer protein PrdC form 2	TIGR04481	UPGQ	1	WP_245122565.1	(61, 62)
Multiheme c-type cytochrome protein ExtKL (cytochrome c family protein)	NF040886	–	1	WP_275805277.1	(61, 62)
GSU2204 family CXXCH-containing protein (MtrB/PioB family outer membrane beta-barrel protein)	NF041027	–	1	WP_272940843.1	(61)
UshA-like protein	NF041198	CxU	1	WP_263046620.1	(61)
C-GCAxxG-C-C family protein form 2	PF09719	–	1	WP_012158890.1	(61)
Mercury methylation corrinoid protein HgcA (CO dehydrogenase/acetyl-CoA synthase gamma subunit)	NF040863	CU	1	WP_250697417.1	(61, 63)
YeeE/YedE thiosulfate transporter family protein	PF04143	GUxSG	1	WP_012471001.1	(61, 62)
UGC-containing TlpA disulfide reductase family protein	cd02966	UGC	1	MDE0950781.1	(61, 62)
Ferredoxin-thioredoxin reductase	NF041072	CxU	2	MCS5647748.1	(61)
Putative regulatory protein, FmdB family	TIGR02605	UxxU	2	–	(61)
Trx7/PDZ domain-containing protein	NF041199	CxxU	1	MCX6587993.1	(61)
Hypothetical protein GOS_B (GOS metagenome)	NF041374	–	1	NBR19009.1	(61)
Hypothetical protein GOS_C (GOS metagenome)	NF045500	UxxC	1	MDP6169091.1	(61)
Redoxin family protein	PF08534	UxxC	1	HEU0032618.1	(61, 62)
DsbA-like protein form 2 (crotonase/enoyl-CoA hydratase family protein)	PF01323	DxxUP	1	MDP6049977.1	(62)
Cobalamin-binding protein BtuF	cd01144	CxxU	1	MDE0719563.1	(62)
Cys-Cys-COOH selenoprotein SaoC	NF040734	CU	1	WP_252346668.1	(63)
ABC transporter substrate-binding subunit SaoX	NF040735	UxP	1	WP_248000900.1	(63)
Mercury methylation ferredoxin HgcB	NF040864	GVGU	1	WP_250697400.1	(63)
ABC transporter substrate-binding selenoprotein SaoB	NF040727	CUxxT	1	WP_242649186.1	(63)
ABC transporter permease subunit SaoP	NF040733	–	1	WP_242649185.1	(63)
Thioredoxin-like selenoprotein SaoT	NF040730	UxxC	1	WP_240067706.1	(63)
UXX-star selenoprotein family 2	NF041211	GxGU	1	WP_366533864.1	(64)
Thiol peroxidase Prx-SUH	NF045827	CxxU	1	WP_246863588.1	(64)
Methyltransferase domain-containing selenoprotein MduS	PF13649	CxxU	2	WP_332845911.1	(64)
HUGP/GUXX-star fusion selenoprotein	NF041212	–	2	WP_318581321.1	(64)
Methyltransferase/UXPK-star double selenoprotein	NF047649	UxxG	2	WP_391856372.1	(64)
Metal-binding double selenoprotein MbdU	NF045648	CU	2	WP_327229162.1	(64)
Arsenite methyltransferase/DUF2284 domain selenoprotein	NF041536	UxxG	2	WP_272482928.1	(64)
Arsenite methyltransferase/AhpD domain selenoprotein	NF041537	UxxG, CxxU	3	WP_349247334.1	(64)
Radical SAM selenoprotein TrsS	NF045646	–	1	WP_327020591.1	(64)
LULAXC motif selenoprotein TsoA	NF045695	LULAxC	1	WP_332113294.1	(64)
Rhodanese-like domain-containing protein form 5 (rhodanese/DsbD fusion-like selenoprotein TsoB)	NF045694	UxxxxU	2	WP_332113227.1	(64)
NEPxGxxU motif selenoprotein TsoC	NF045708	GxxU	1	WP_332369869.1	(64)
HSGNPxU motif selenoprotein TsoX	NF045809	UxxC	1	WP_337661155.1	(64)
Selenoprotein TsoY	NF047643	UxxV	1	WP_376797693.1	(64)
HUIPC motif thioredoxin-like selenoprotein	NF047660	UxxC	1	WP_393970542.1	(64)
TonB-dependent receptor, FCYXU motif-type	NF049945	CxxU	1	WP_419836696.1	(65)
Selenoprotein O-like protein	PF02696	–	1	WP_136798779.1	(65)
CTP synthase C-terminal region-related selenoprotein (glutamine amidotransferase)	NF004836	GxUxG	1	WP_419249736.1	(65)
Type III sulfide quinone reductase, selenoprotein subtype (NAD(P)/FAD-dependent oxidoreductase)	NF049943	HxxU	1	MGW8192990.1	(65)
DUF523 domain-containing protein	PF04463	SxSUG	1	WP_419761712.1	(65)
Hypothetical protein DG	NF049953	GUxG	1	WP_419249779.1	(65)
Hypothetical protein DW	NF041197	GxU	1	SMF39960.1	(65)
Hypothetical protein DF	NF049942	–	1	WP_419720682.1	(65)
Thioredoxin-like selenoprotein Sec.1	PF13192	CxU	1	WP_232817751.1	(71)
Thioredoxin-like selenoprotein Sec.2	PF13192	UxC	1	WP_218069652.1	(71)
KCU-star family selenoprotein (or DUF466 protein)	NF033934	CU	1	WP_052061029.1	(89)
Selenobacteriocin	TIGR04081	SGUG	2	WP_074187118.1	(90)
SO_0444 family Cu/Zn efflux transporter	NF033936	UxC	1	WP_014809615.1	GA^*c*^
Tetrathionate reductase family octaheme c-type cytochrome	TIGR04315	–	1	WP_331457571.1	GA
UshA-like selenoprotein family 2	NF049981	CxxU	1	WP_420705249.1	GA
Nitrogenase component I subunit alpha	TIGR01862	RxU	1	WP_153802866.1	GA

^*a*^ Selenoprotein families found in both bacteria and archaea.

^*b*^ –, not available.

^*c*^ GA, GenBank annotated.

Formate dehydrogenase alpha subunit (FdhA) stands out as one of the earliest enzymes confirmed to contain Se and the Sec-UGA codon in its gene sequence and is the most widely distributed selenoprotein family among bacteria (16, 91). These enzymes catalyze the two-electron oxidation of formate to carbon dioxide (CO_2_) in diverse metabolic pathways (92). In the active site of FdhA, the Se atom in Sec is directly coordinated with a metal cofactor (molybdenum (Mo) or tungsten (W)) that is necessary for enzymatic activity. Although the exact role of Se in FDH-catalyzed reactions remains unresolved, it is postulated to stabilize transient oxidation states. This characteristic results in a relative difficulty in achieving higher oxidation states and facilitates the non-enzymatic reduction of these enzymes, which is advantageous for organisms whose lifestyle makes their FDHs more prone to suffer oxidative modifications (93). In addition, methanogenic archaea contain formylmethanofuran dehydrogenase subunit B (FwdB), a distant homolog of FdhA. FwdB catalyzes the reduction of CO₂ to formate, which subsequently condenses with methanofuran to form formylmethanofuran (94). Like FdhA, the functional significance of Se in FwdB-mediated catalysis is unknown as well. Previous comparative genomic analyses of prokaryotic selenoproteins revealed that FdhA is often the sole selenoprotein in many bacterial species, suggesting its potential role in sustaining Sec utilization and metabolic adaptability in these organisms (62). However, the limited detection of FdhA homologs in multiple large-scale marine metagenomic data sets implies the presence of alternative selenoproteins (such as SelD) that may independently maintain the Sec utilization trait under distinct ecological pressures (59, 61).

SelD is the second most expansive prokaryotic selenoprotein family that catalyzes the synthesis of selenophosphate, the biologically activated form of Se essential for both selenoprotein biosynthesis and other Se-dependent metabolic pathways (95). These proteins feature Sec as a catalytic residue in their N-terminal flexible Se-binding loop. Although free selenide is frequently used as the Se substrate for *in vitro* selenophosphate synthesis (96), the actual *in vivo* form of Se utilized by SelD and the corresponding donor system responsible for its delivery remains incompletely elucidated. Research has proposed that various machineries, including the glutathione (GSH) and Trx systems, Sec lyase, Cys desulfurase, and Se-binding proteins, may be involved in the delivery of Se to SelD (41). Additionally, several SelD-like proteins identified in hyperthermophilic S-reducing archaea (such as *Sulfolobales* and *Thermoproteales*) are speculated to participate in archaeal S metabolism (97).

Glycine reductase (GR) catalyzes the reductive deamination of glycine, a reaction coupled to the esterification of acetyl-phosphate, ultimately leading to ATP formation (98, 99). GR is composed of three subunits—protein A (GrdA), protein B (GrdB), and protein C (GrdC)—with GrdA and GrdB functioning as selenoproteins in many organisms. The substrate-binding subunit GrdB binds glycine and is responsible for the release of NH_3_, while GrdA contains glycine that is reduced by Trx, generating an intermediate utilized by GrdC to produce acetyl-phosphate. GrdA likely interacts with the UxxCxxC motif of GrdB, and the CxxU motif in GrdA itself exhibits redox activity. Together, this enzyme complex allows certain anaerobic bacteria to conserve energy through a soluble substrate-level phosphorylation pathway (99).

D-proline reductase has been characterized as a membrane-associated homodecameric enzyme. In some bacteria, such as clostridia, the 26-kDa subunit (PrdB), which shares homology with GrdB, contains Sec (82, 100). This enzyme plays a role in amino acid metabolism, specifically catalyzing the reductive ring cleavage of D-proline to produce 5-aminovalerate. The reductive deamination of amino acids by this enzyme is analogous to the reaction catalyzed by GR; however, unlike GR, such a reduction process is not coupled with substrate-level phosphorylation (6). Nevertheless, it has been suggested that proline reduction may contribute to energy conservation through a chemiosmotic mechanism (82).

Nickel-iron [NiFe] hydrogenase is one of the three major classes of hydrogenases, which contains a [NiFe] center and catalyzes the reversible dissociation of molecular hydrogen (H_2_) into protons and electrons (101). Certain [NiFe] hydrogenases have been identified to contain Sec (forming a subclass known as [NiFeSe] hydrogenases), such as coenzyme F420-reducing hydrogenase (alpha and delta subunits, FrhA and FrhD), methylviologen-reducing hydrogenase alpha subunit (MvhA/VhuA/VhuU), and F420-non-reducing hydrogenase delta subunit (MvhD/VhuD) (76–78, 102, 103). They are exclusively found in sulfate-reducing and methanogenic microorganisms. The mechanism underlying [NiFeSe] hydrogenase activity is complex, but the specific advantage of Sec seems to be related to its ability to protect Ni in the active site from irreversible overoxidation. It has been proposed that the Sec residue is coordinated to Ni, and the heterolytic cleavage of H_2_ is mediated by the combined action of the Ni center and the Sec residue (104, 105). Furthermore, multiple Sec residues have been observed in some Sec-containing FrhD/VhuD proteins in archaea (37).

Heterodisulfide reductase (HDR) is an Fe-S cluster-containing protein that catalyzes the formation of coenzyme M (CoM-SH) and coenzyme B (CoB-SH) by the reversible reduction of the heterodisulfide (CoM-S-S-CoB) ( 81, 106). This reaction plays a critical role in recycling the two thiol coenzymes required for the final step of microbial methanogenesis. HDR consists of three distinct subunits, with the alpha subunit (HdrA) containing Sec. In methanogenic archaea such as *M. maripaludis*, the electrons for this reaction come from either formate or H_2_ via FDH or HDR-associated hydrogenase Vhu. VhuD appears to be central to the interaction of both enzymes with HdrA, as the genes encoding VhuD and HdrA are often fused in archaeal genomes (78).

Peroxiredoxins (Prxs) are a ubiquitous family of antioxidant enzymes that reduce intracellular H₂O₂ to maintain redox homeostasis and mediate cellular signaling (107). These proteins are present in virtually all organisms, with certain bacteria expressing Sec-containing Prx variants (83). A highly conserved PxxxTxxU motif in these enzymes is crucial for hydrogen bonding with the peroxide substrate, thereby facilitating their high catalytic efficiency (108).

Trxs comprise a class of small and highly conserved thiol-disulfide oxidoreductases that function as antioxidants to protect cells from the harmful effects of free radicals. These proteins are universally distributed in all organisms from bacteria to humans, serving as essential redox regulators in cellular homeostasis. Certain bacteria have the Sec-containing forms of Trx, where a UxxC catalytic motif in their active sites is specifically adapted to work efficiently under high substrate concentrations (12, 84).

Glutaredoxins (Grxs) are small oxidoreductases that reduce disulfide bonds in proteins using GSH as a redox cofactor. Sec-containing Grx proteins have been identified in a limited number of bacterial species (85, 109). These enzymes exhibit a characteristic Trx-like fold and feature a conserved U/CXXC/S active-site motif in their N-terminal regions. Similar to Trxs, the incorporation of Sec significantly enhances the catalytic efficiency of these thiol oxidoreductases (85).

MsrA is an important protein repair enzyme that specifically reduces methionine-*S*-sulfoxide back to methionine, thereby playing an essential role in protecting cells against oxidative damage (110). The general catalytic mechanism of MsrA comprises three steps: (i) the catalytic Cys residue attacks the S atom of methionine sulfoxide, leading to the formation of a Cys sulfenic acid intermediate and the concurrent release of methionine; (ii) the catalytic Cys sulfenic acid interacts with a resolving Cys residue to establish an intramolecular disulfide bond; (iii) the disulfide bond is reduced by cellular reductants such as Trx to restore enzyme activity (111). Interestingly, in certain bacteria, MsrA exists as a selenoprotein where the catalytic Cys is replaced by Sec, forming a GUFWG/H active-site motif. This substitution underscores the significant contribution of Se to the catalytic efficiency of this selenoenzyme (86, 112, 113).

Several transporter subunits and associated proteins have been predicted to contain Sec residues, such as putative mercuric transport protein, cation-transporting ATPase E1-E2 family, YeeE/YedE thiosulfate transporter family protein, ABC transporter substrate-binding subunits SaoX and SaoB, ABC transporter permease subunit SaoP, Cys-Cys-COOH selenoprotein SaoC, and SO_0444 family copper/zinc (Cu/Zn) efflux transporter (60–64). Most of these selenoproteins are implicated in heavy metal or metalloid resistance (e.g., mercury [Hg] and arsenic [As]). In selenoproteins such as SaoB, SaoC, and the UXX-star selenoprotein family, Sec residues may function as metal-binding ligands or interact with other unknown substrates (63, 64). However, their precise roles in heavy metal detoxification pathways require further investigation.

Other prokaryotic selenoproteins include arsenite methyltransferase, HesB-like protein, deiodinase-like protein, GPX-like protein, SELENOW-like protein, Fe-S oxidoreductase-like protein, DsrE-like protein, arsenate reductase, disulfide bond formation protein A (DsbA)-like protein, glutathione S-transferase (GST)-like protein, UGSC-containing protein, and a variety of Prx-/Trx-/Grx-like proteins that exhibit a Trx-like fold. As previously discussed, nearly all of these selenoproteins have been identified via robust bioinformatics approaches; however, their biological functions remain largely uncharacterized. By using SitesBLAST (114), a tool designed to infer functional roles of conserved residues (including Sec) in proteins based on experimental studies of their homologous proteins, we found that approximately 20% of known selenoprotein families exhibit predicted binding capacity for diverse atoms or chemical moieties, with ~70% of these interactions involving metals (such as Mo, Fe, Zn, Hg, and As) or metal-derived molecules (such as Fe-S clusters). These results strongly suggest that Se plays a critical role in metal homeostasis and enzymatic function. However, with the exception of a limited number of well-characterized selenoproteins (e.g., FdhA, FrhA, and FrhD), all predictions were inferred from experimental evidence of their Cys-containing homologs. Therefore, future studies are needed to confirm direct Sec-ligand interactions and clarify their physiological roles.

## Sec VERSUS Cys

It is widely acknowledged that Sec generally exhibits superior enzymatic efficiency and faster reaction kinetics compared to Cys, primarily due to the unique chemical properties of its selenol group. The selenol’s significantly lower pKa value (5.2 vs. 8.3 for Cys thiol) renders it more deprotonated at physiological pH, enhancing nucleophilicity and facilitating electron/proton transfer reactions (115, 116). This is further supported by the weaker Se–H bond, which makes Sec less basic than Cys and promotes more efficient redox cycling during catalysis (93). These advantages are critical for the high-performance activity of antioxidant enzymes (such as GPX) where Sec enables rapid reduction of substrates while resisting irreversible oxidation. Additionally, Se may bind to thiol groups in proteins to form less toxic Se–S complexes, enhancing cellular adaptability to Se stress (117). However, despite these functional benefits, nearly all selenoenzymes have far more abundant Cys-containing homologs across diverse organisms, suggesting that catalytic efficiency and protective advantages alone cannot fully explain the evolutionary pressure to retain Sec. Thus, the evolutionary dynamics between selenoproteins and their Cys-containing counterparts require thorough investigation to elucidate the selective forces driving Sec preservation.

## SELENOPROTEIN OPERON

Although selenoprotein families generally exhibit limited sequence homology and participate in diverse biological processes, co-localized selenoprotein genes within operons often function synergistically in the same pathway. Typical examples are the *grdA-grdB*, *prdB-prdC*, and *frhA-frhD-hdrA* operons. GrdA and GrdB are key components of GR and are frequently organized as a single operon (81), while PrdB and the proline reductase-associated electron transfer protein PrdC are involved in the D-proline reductase mechanism (88). The *frhA*, *hdrA*, and *frhD* genes are frequently grouped together in archaea and have also been found clustered in some bacteria, particularly *Deltaproteobacteria*, where an additional selenoprotein gene encoding the Fe-S oxidoreductase-like protein (GlpC) exists to form a four-selenoprotein-gene operon (56, 118). Although FrhA and FrhD are known to be functionally related, their interactions with the other two selenoproteins remain unclear. Comparative genomics indicates potential horizontal transfer of the entire *frhA-frhD-hdrA* operon between archaea and *Deltaproteobacteria*, implying a complex and highly dynamic evolutionary trajectory of these selenoproteins in prokaryotes (118). In addition, metagenomic studies have revealed operonic clustering of selenoprotein genes for some other oxidoreductases, such as Prx and SELENOW-like proteins, as well as Prx-like and AhpD-like proteins (61). Although the exact functions of these selenoproteins are unknown, the genomic colocation of their genes suggests that they might be involved in common redox-related processes.

Very recently, larger selenoprotein operons have been identified in specific bacterial lineages, including the SAO (Sec-assisted organometallic) system operon (containing six selenoprotein genes) and the Tso (three selenoprotein operon) operon (containing three selenoprotein genes along with multiple adjacent yet otherwise unrelated selenoprotein genes) (63, 64). This co-operonic organization suggests a new evolutionary strategy where the biochemical advantages of Sec incorporation are maximized and preserved through pathway-level coordination, especially in specialized metabolic pathways requiring multiple enzymatic steps with precise electron flux regulation.

## EVOLUTION OF Sec-ENCODING MACHINERY, SELENOPROTEINS, AND SELENOPROTEOMES

Previous comparative genomics studies have examined the distribution and evolutionary patterns of Sec-encoding machinery and selenoproteins across diverse bacterial and archaeal lineages, providing a broad understanding of the status of Sec utilization and function in prokaryotes (16, 53, 62, 72, 89, 119–121). It seems that Sec utilization represents an ancient trait that was once prevalent among the majority of bacterial clades but has undergone selective retention in specific proteins or acquisition in distinct species during evolution. It has been proposed that many bacterial selenoproteins (especially thiol-based oxidoreductases) originated from their widespread Cys-containing homologs through Cys-to-Sec conversions, wherein the catalytic Cys residues were replaced by Sec (118). This phenomenon reflects Sec’s superior catalytic efficiency in redox reactions despite the inherent risks of increased reactivity and potential toxicity. In contrast, Sec-to-Cys substitutions remain rare, with documented cases primarily limited to FdhA and SelD. Such a bidirectional evolutionary pattern suggests a dynamic balance between selenoprotein innovation and functional regression to Cys-containing homologs, potentially driven by environmental Se availability or metabolic demands. This trend is opposite to eukaryotes, where Sec-to-Cys replacements appear to be more common (25). Such a disparity is likely due to the easier *de novo* acquisition and greater plasticity of SECIS elements in bacteria compared to eukaryotes. The bacterial selenoproteome varies widely among different organisms, encompassing distinct sets of protein families. The organisms most enriched with selenoprotein genes are predominantly anaerobic, such as *Thermodesulfobacteriota* (formerly *Deltaproteobacteria*), *Synergistota*, and *Clostridia*. Previously, the largest bacterial selenoproteome was found in *Olavius algarvensis* Delta 1 endosymbiont, a symbiotic bacterium isolated from the marine gutless worm *O. algarvensis*, with at least 57 selenoprotein genes (60). Very recently, a representative MAG from the *Methylomirabilota* phylum was predicted to contain 63 selenoprotein genes, suggesting a new upper bound for bacterial selenoproteome size despite the potential for multiple closely related species or strains to be co-assembled in the same MAG (66). Although the mechanisms driving such an unusual distribution of Sec utilization remain unresolved, the observed trade-off between Sec acquisition and selenoprotein loss events may partially account for the discrepancy between the catalytic advantages provided by Sec and its limited use in nature (72). Moreover, hypoxic or anaerobic environments appear to have facilitated the evolution of novel selenoprotein genes (62). Bacterial selenoproteins predominantly participate in anaerobic metabolic pathways, where the superior nucleophilicity of Sec may enhance catalytic efficiency under reducing conditions (6). In aerobic environments, however, organisms are unable to tolerate the high reactivity of Sec, as its selenol group is highly susceptible to oxidation and subsequently promotes the production of reactive oxygen species, thereby threatening cellular redox homeostasis. Consequently, aerobic bacteria rarely retain selenoproteins, as the metabolic cost of maintaining Sec exceeds its catalytic benefits under oxidative conditions.

In archaea, the capacity to incorporate Sec into proteins is constrained to a limited number of lineages, including the orders *Methanococcales*, *Methanopyrales*, and the Asgard superphylum (32, 53–55, 72). A total of 11 distinct selenoprotein families have been identified in archaea so far (Table 1). Among them, SelD, FrhA, MvhA/VhuA/VhuU, FrhD/MvhD/VhuD, and HdrA were detected in nearly all Sec-utilizing archaea, suggesting their essential roles in Se-dependent metabolism. In contrast, FdhA, the most widespread selenoprotein in bacteria, is restricted to specific species in *Methanococcales* and *Methanopyrales* (56, 72). On the other hand, three additional families—Prx-like protein, homolog of the AhpF N-terminal (Grx-like) domain, and DsbA-like protein—were exclusively found in Asgard archaea (56). In the Asgard superphylum, the majority of sequenced organisms from the clades *Lokiarchaeia/Helarchaeales*, *Thorarchaeia*, and *Sifarchaeia/Borrarchaeales* utilize Sec, whereas other clades appear to lack this ability. The selenoproteomes reported in archaea show a relatively narrow distribution, ranging from 1 to 19 selenoproteins, with the *Lokiarchaeia/Helarchaeales* and *Sifarchaeia/Borrarchaeales* clades generally possessing larger selenoproteomes compared to other archaea (56). The *Lokiarchaeota* archaeon isolate bin41, isolated from ancient Siberian permafrost sediment (122), currently holds the record for the largest known archaeal selenoproteome, with 19 selenoprotein genes identified in its MAG (56). However, since nearly all Asgard archaeal genomes are reconstructed from metagenomic data, these MAGs may represent mixtures of closely related species or strains. Further efforts are needed to obtain more accurate genomic sequences for Asgard archaea.

Although archaea and eukaryotes share a conserved Sec-encoding machinery, suggesting an archaeal origin for the eukaryotic Sec incorporation system, the canonical eaSECIS elements and eSECIS elements show no sequence or structural similarities. However, recent discoveries of eSECIS-like structures in several Asgard archaea provide evidence for intermediate forms linking archaeal and eukaryotic Sec-encoding systems (53–55). Despite these findings, the precise mechanisms underlying the evolutionary transition of this machinery from archaea to eukaryotes remain unclear. Huang et al. conducted a comprehensive analysis of Sec machinery in all known Sec-utilizing archaea, with a particular focus on hundreds of Asgard archaeal species (56). This work generated the most detailed and systematic map to date of Sec utilization in this evolutionarily significant archaeal superphylum. Notably, investigation of eSECIS-like structures in archaeal selenoprotein genes revealed that most Asgard archaeal selenoprotein genes contain eSECIS-like elements with varying degrees of sequence and structural diversity. These findings support the hypothesis that canonical eaSECIS elements may have evolved from Asgard archaeal SECIS elements via horizontal gene transfer, indicating a complex and dynamic evolutionary history of SECIS elements in archaea. Based on these observations, a plausible evolutionary roadmap has been proposed for the transition of SECIS elements from archaea to eukaryotes, with SelD being identified as a potential intermediate in the emergence of ancestral eSECIS elements. These insights enhance our understanding of the macro-evolutionary dynamics of the Sec biosynthetic pathway across the tree of life, highlighting the role of Asgard archaea as a critical bridge between prokaryotic and eukaryotic Sec systems. Further studies are warranted to elucidate the mechanistic and evolutionary details of the archaea-to-eukaryote transition of both Sec-encoding machinery and selenoproteins with the increasing number of new archaeal genomes, especially from the Asgard superphylum.

## CONCLUDING REMARKS

The identification and characterization of selenoprotein genes in genomic sequences are pivotal for unraveling the mechanisms of Se utilization and its functional roles in diverse organisms. This endeavor has been significantly advanced by the development and application of robust and highly efficient computational methodologies. Over the past decade, particularly in the last 2–3 years, more than 110 selenoprotein families or subfamilies have been reported, with the majority belonging to thiol-based oxidoreductases. This review focuses on the recent progress achieved in the *in silico* identification of selenoproteins in prokaryotes. Additionally, it presents comparative genomic investigations concerning Sec-encoding machinery, selenoproteins, and selenoproteomes in bacteria and archaea. These studies have provided valuable insights into the general principles of Sec utilization and the evolutionary trajectories observed in the natural world. With the exponential growth of genomic data and rapid advancements in computational techniques for detecting selenoprotein genes, bioinformatics, comparative genomics, and integrative multi-omics approaches will play an even more significant role in elucidating Se utilization and function in nature.
